# Inflammation Enhances IL-2 Driven Differentiation of Cytolytic CD4 T Cells

**DOI:** 10.1371/journal.pone.0089010

**Published:** 2014-02-20

**Authors:** Aspen M. Workman, Ashley K. Jacobs, Alexander J. Vogel, Shirley Condon, Deborah M. Brown

**Affiliations:** 1 School of Biological Sciences, University of Nebraska-Lincoln, Lincoln, Nebraska, United States of America; 2 Nebraska Center for Virology, University of Nebraska-Lincoln, Lincoln, Nebraska, United States of America; St. Jude Children’s Research Hospital, United States of America

## Abstract

Cytolytic CD4 T cells (CD4 CTL) have been identified *in vivo* in response to viral infections; however, the factors necessary for driving the cytolytic phenotype have not been fully elucidated. Our previously published work suggests IL-2 may be the master regulator of perforin-mediated cytotoxicity in CD4 effectors. To further dissect the role of IL-2 in CD4 CTL generation, T cell receptor transgenic mice deficient in the ability to produce IL-2 or the high affinity IL-2 receptor (IL-2Rα, CD25) were used. Increasing concentrations of IL-2 were necessary to drive perforin (Prf) expression and maximal cytotoxicity. Granzyme B (GrB) expression and killing correlated with STAT5 activation and CD25 expression *in vitro*, suggesting that signaling through the high affinity IL-2R is critical for full cytotoxicity. IL-2 signaling was also necessary *in vivo* for inducing the Th1 phenotype and IFN-γ expression in CD4 T cells during influenza A (IAV) infection. In addition, GrB expression, as measured by mean fluorescent intensity, was decreased in CD25 deficient cells; however, the frequency of CD4 cells expressing GrB was unchanged. Similarly, analysis of cytolytic markers such as CD107a/b and Eomesodermin indicate high IL-2Rα expression is not necessary to drive the CD4 CTL phenotype during IAV infection. Thus, inflammatory signals induced by viral infection may overcome the need for strong IL-2 signals in driving cytotoxicity in CD4 cells.

## Introduction

CD4 T cells play a central role in immune responses to infection as well as acting in a regulatory role for maintaining homeostasis. During activation, CD4 T cells are instructed by the cytokine environment to differentiate into one of several distinct subsets of T helper (Th) cells [1]. Viral infections typically induce the Th1 polarized subset that secretes predominantly IFN-γ, induces macrophage activation, helps B cells make IgG2a antibodies and promotes CD8 T cell function and memory [2]. CD4 T cells can play an additional role in viral clearance by supplementing their helper function with cytotoxicity. MHC class II restricted CD4 effectors with cytolytic potential have been described since the late 1970s [3] and while early reports confined this activity to *in vitro* stimulated CD4 effectors [4]–[6], recent data underscores this cell type as an important mediator of viral clearance (reviewed in [7]–[9]). Cytolytic CD4 T cells (CD4 CTL) have been identified in humans with chronic infections such as Epstein-Barr Virus [10], cytomegalovirus [11] and Human Immunodeficiency Virus [12], suggesting prolonged exposure to antigen induces a terminally differentiated effector capable of cytotoxic activity. CD4 CTL have also been described during acute viral infections such as influenza [13], [14], LCMV [15], and ectromelia virus [16]. Demonstration of CD4 cytolytic activity in these infections suggests CD4 cells have a more direct role in viral clearance than was previously appreciated [13], [14], [16].

Early mechanistic studies revealed that CD4 CTL killed in a manner that was dependent on the expression level of Fas on the target cell, where increased cytotoxicity correlated with increased Fas expression [17]. However, in many of the early studies, CD4 CTL were generated using potent T cell mitogens such as anti-CD3 or concanavalin A (ConA) for activation [17]–[19]. In more recent studies, CD4 CTL generated *in vivo* in response to infection or vaccination and characterized *ex vivo* demonstrated that perforin mediated cytotoxicity was the dominant mechanism utilized by CD4 CTL [11]–[13], [20], [21]. Indeed, recent studies utilizing *in vivo* cytotoxicity assays indicate that CD4 CTL are potent, expeditious killers approaching CD8 T cell efficiency when analyzed on a per cell basis [22]. Our lab has demonstrated previously that *in vitro* generated CD4 effectors could lyse targets via FasL and perforin mediated mechanisms and this was dependent on peptide concentration and exogenous IL-2 [23]. In contrast, *in vivo* generated effectors used perforin exclusively to mediate cytotoxicity after activation by influenza viral infection [13], ectromelia virus [16] and in anti-tumor vaccination models [24].

Until recently, little was known about the signals that drive the differentiation of CD4 CTL, especially from naive CD4 T cell populations. Recent data using anti-tumor models has demonstrated that strong stimulation via OX40 and OX40-L [24], 4-1BB activation [25] or OX40 stimulation and lymphopenia [26] could enhance CD4 CTL activity in a manner dependent on Eomesodermin. In addition, recent reports suggest inflammatory signals such as type I interferons cooperate with IL-2 receptor signaling *in vivo* to promote CD4 CTL activity [27]. Our lab has made use of T cell receptor (TCR) transgenic (tg) CD4 T cells specific for a single peptide to allow for the delineation of the factors responsible for the generation of the cytolytic phenotype *in vitro* and *in vivo*. Our previous work has demonstrated that Th1 (IL-2, IL-12 and αIL-4) but not Th2 (IL-2, IL-4, and αIFN-γ) polarizing conditions lead to the generation of cytolytic CD4 cells that express granzyme B (GrB) and can kill peptide pulsed targets [23], [28]. We further demonstrated that exogenous IL-2 was necessary and sufficient to induce a cytolytic phenotype in CD4 T cells stimulated *in vitro* with peptide pulsed antigen presenting cells (APC) [23]. These studies did not address the contribution of endogenous IL-2 produced by CD4 effectors, or the role of IL-2 signaling in driving CD4 CTL differentiation *in vivo*. Therefore, in this study, we used ovalbumin (Ova) specific TCR transgenic mice in which either IL-2 or the high affinity receptor for IL-2 (IL-2Rα, CD25) was deleted to determine the relative contribution of IL-2 signaling to the development of CD4 T cells with cytolytic activity.

In this report, we found that IL-2 regulates GrB and perforin (Prf) expression in a dose dependent manner *in vitro*. IL-2, but not other common γ chain cytokines induced sustained STAT5 activation that was necessary for GrB expression and killing activity. In addition, the level of IL-2Rα correlated with maximal CTL activity *in vitro* and maximal GrB expression *in vivo*. Similarly, we found that IL-2 signaling was required *in vivo* for maximal GrB expression in response to influenza virus infection. However, expression of cytolytic markers and degranulation was independent of high IL-2Rα levels during IAV infection. These data highlight the importance of IL-2 in driving the differentiation of CD4 cells with cytolytic potential when inflammation is limiting. However, inflammatory cytokines induced during infection may enhance cytotoxicity independent of IL-2R signaling. This information will be important when designing vaccine strategies against emerging viral infections and for influenza viruses with pandemic potential.

## Results

### IL-2 Regulates Perforin Expression and Cytotoxicity in a Dose Dependent Manner in vitro

Our earlier work demonstrated that exogenous IL-2 and peptide pulsed antigen presenting cells (APCs) were the minimal requirements necessary to induce perforin (Prf)-mediated cytotoxicity in CD4 T cells [23]. However, given that IL-2 is one of the first cytokines produced by CD4 T cells upon TCR stimulation and is used in an autocrine fashion to initiate cell cycle progression and proliferation [29], it was difficult to determine the strength and duration of signal necessary to induce cytotoxicity in CD4 cells. To overcome this problem, the present study used Ova peptide specific TCR tg mice deficient in the ability to produce IL-2 (DO11.10/IL-2^−/−^; [30]), allowing us to control the amount of IL-2 in each experiment.

**Figure 1:**
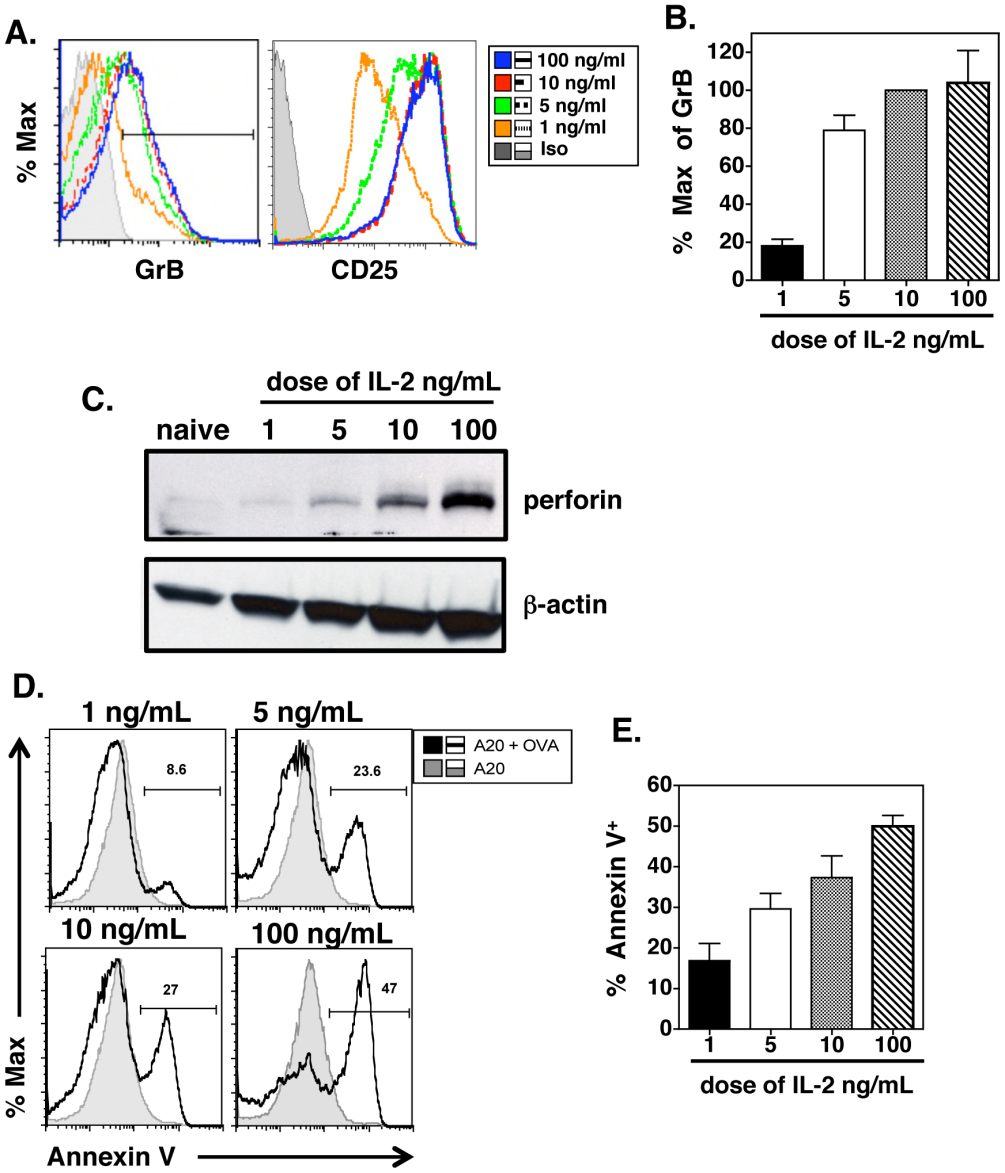
IL-2 regulates perforin expression and cytotoxicity in a dose dependent manner *in vitro*. CD4 effectors were generated from naive DO11.10/IL-2^−/−^ Ova specific TCR tg CD4 cells as described in the Materials and Methods using peptide pulsed APC and increasing doses of IL-2. Four days later, cells were collected and stained for CD4, KJ126 (Ova specific TCR), CD25 and intracellular levels of GrB (**A**). The histograms show levels of GrB and CD25 expression on gated CD4^+^/KJ126^+^ effectors generated with increasing doses of IL-2. **B**) The percent of maximum GrB expression was calculated by dividing the GrB MFI in CD4 cells incubated with 1, 5 or 100 ng/ml IL-2 by the GrB MFI in CD4 cells incubated with 10 ng/ml as the control. Shown is the average +/− SD of 3–4 separate experiments. **C**) Freshly isolated naive TCR tg CD4 cells, or CD4 effectors grown in the presence of increasing IL-2 concentration were resuspended in lysis buffer as described. Cell lysate was run on a 7.5% polyacrylamide gel, transferred to membrane and perforin protein detected with mouse anti-perforin antibody. A 66 kD band is shown for perforin (top panel) and β-actin (bottom panel) is shown as a loading control. **D**) CD4 effectors were analyzed for their ability to lyse target cells by co-incubation with Ova peptide pulsed A20 cells for 4 h at a 3∶1 E:T ratio. Target cells were then stained with antibody to Annexin V as a marker of target cell killing. Percent Annexin V positive cells in each group was determined after gating on CD4-negative A20 cells in the presence (open histograms) or absence (shaded histograms) of Ova peptide. Representative histograms are shown with numbers indicating percent Annexin V^+^ A20 cells pulsed with peptide after gating based on negative control (unpulsed A20 cells). Panel (**E**) shows the average +/− SD of percent Annexin V positive cells at each dose of IL-2 from 3–4 independent experiments.

To elucidate the contribution of IL-2 in inducing cytotoxicity, naive DO11.10/IL-2^−/−^ CD4 T cells were cultured for 4 days in the presence of Ova peptide pulsed APCs and increasing concentrations of IL-2. Figure 1A demonstrates that exogenous IL-2 induces granzyme B (GrB) and CD25 expression in a dose dependent manner, however, maximal GrB expression is apparent at 5–10 ng/ml IL-2 while 100 ng/ml IL-2 does not further enhance GrB protein expression (Fig. 1B). To determine whether IL-2 also induced perforin (Prf) expression in CD4 T cells, we measured Prf protein by western blot analysis in the presence of increasing concentrations of IL-2. Figure 1C demonstrates that Prf expression increases with increasing doses of IL-2. Unlike the regulation of GrB, Prf is increased in CD4 effectors cultured with 100 ng/ml IL-2 compared to CD4 cells cultured with 10 ng/ml IL-2. Furthermore, cytotoxicity was found to increase with increasing concentrations of IL-2 (Figure 1D). Shown are representative histograms of Annexin V staining of peptide pulsed target cells incubated with CD4 effectors generated with increasing concentrations of IL-2. Also shown is the average percentage of Annexin V^+^ target cells (Fig. 1E). These results indicate that IL-2 enhances the expression of cytolytic proteins including GrB and Prf and peptide specific cytotoxicity in CD4 effectors. However, it appears that lower doses of IL-2 induce maximal GrB while high dose IL-2 is necessary for maximal Prf expression, suggesting that Prf requires stronger IL-2 signals than GrB.

### Sustained STAT5 Phosphorylation Correlates with GrB Expression and Cytotoxicity

**Figure 2:**
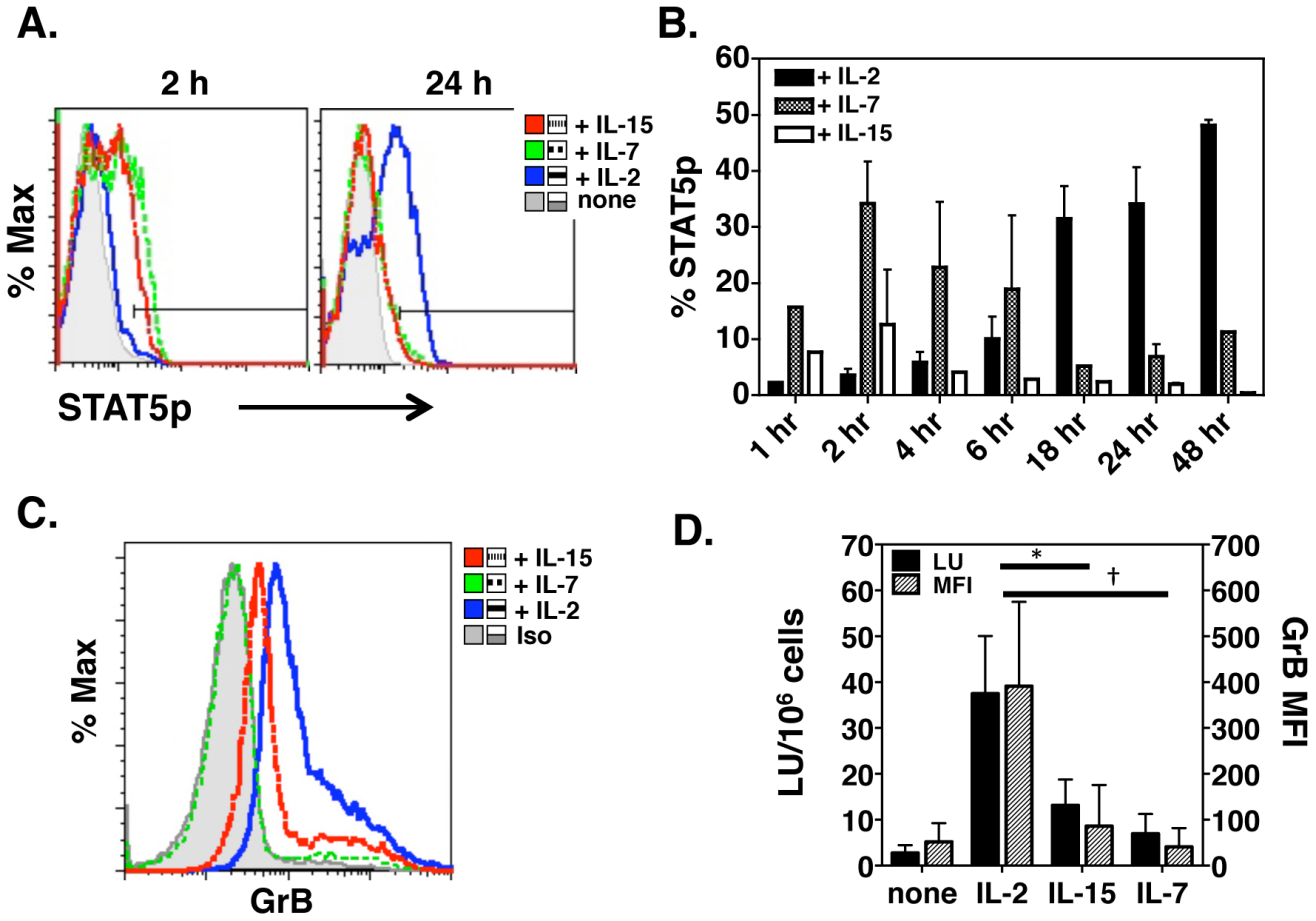
Sustained STAT5 phosphorylation correlates with GrB expression and cytotoxicity. CD4 effectors were cultured with Ova pulsed APCs, 10/ml IL-2, 10 ng/ml IL-7 or 25 ng/ml IL-15. At various time points, cells were collected and stained with antibodies to CD4, KJ126 and anti-phospho STAT5. Representative histograms showing the amount of phosphorylated STAT5 after gating on CD4/KJ126^+^ cells at 2 and 24 h is shown (**A**). Panel B is the average +/− SEM of 3–4 separate experiments of % STATp over time. CD4 effectors grown in IL-2, IL-7 and IL-15 for 4 days were collected and analyzed for GrB expression by flow cytometry (**C**) or killing activity by the JAM assay. This experiment was repeated 4 times and panel **D** is the average +/− SD of lytic units on the left axis compared to MFI of GrB expression on the right axis demonstrating a high correlation between GrB expression and killing activity.

The IL-2 receptor is a heterotrimeric complex consisting of a high affinity cytokine binding subunit (IL-2Rα, CD25) as well as two signaling components, IL-2Rβ and a common γ chain. After IL-2 binds to the receptor complex, JAK3 is activated, leading to the phosphorylation and dimerization of STAT5a and STAT5b [31]. The active STAT5 dimer is then translocated to the nucleus to initiate gene transcription. It has been demonstrated that IL-2 can lead to a direct induction of Prf and GrB gene expression in CD8 CTL through STAT5 activation [32]. To determine if activation of the STAT5 pathway correlated with killing activity in CD4 effectors, we first measured phosphorylation of STAT5 over time. IL-7 (which shares the common γ chain receptor) and IL-15 (which shares the IL-2β and common γ chain receptor) lead to peak STAT5 phosphorylation at 2 hours after culture initiation and these levels diminish over time (Fig. 2A and B). In contrast, IL-2 leads to high levels of STAT5 phosphorylation only after 18 hours (Figure 2B) and these high levels are maintained through 96 hours (data not shown). Although STAT5 is phosphorylated for 2–4 h after IL-7 or IL-15 treatment, this level and/or timing of STAT5 phosphorylation does not induce high levels of GrB (Fig. 2C and 2D). We also tested the ability of these common γ chain cytokines to drive cytotoxicity in CD4 effectors by the JAM assay [23], [28] (Fig. 2C). Percent lysis of peptide pulsed target cells was first converted to lytic units (LU) and averaged, and is shown relative to GrB MFI (Fig. 2D). Cytotoxicity by CD4 effectors incubated with IL-7 or IL-15 is very low and correlates with GrB MFI indicating that other common γ chain cytokines do not promote cytotoxicity.

### Inhibitors of the JAK3/STAT5 Signaling Pathway Inhibit GrB Expression and Killing Activity

**Figure 3:**
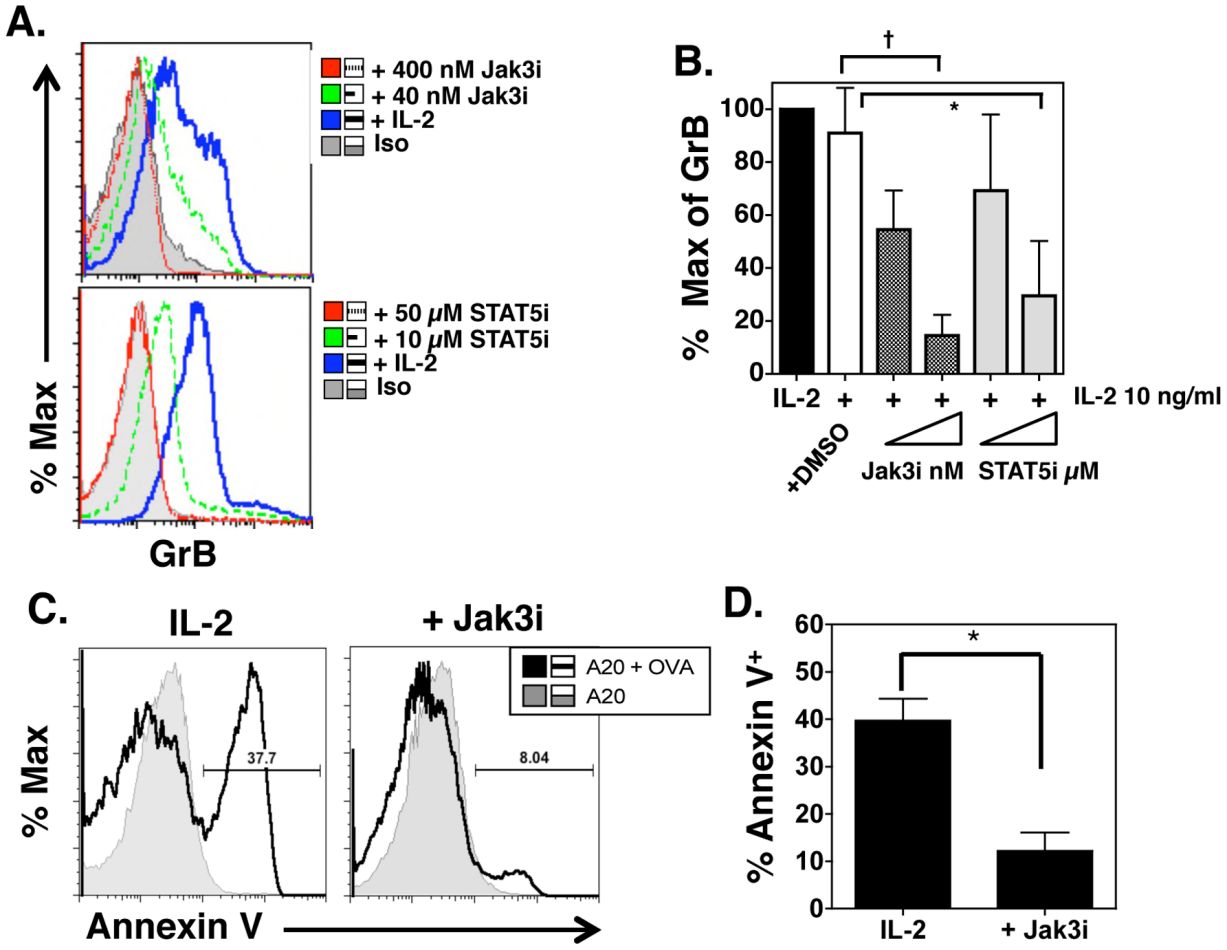
Inhibitors of the Jak3/STAT5 pathway block GrB expression and cytotoxicity. Naive CD4 DO11.10/IL-2^−/−^ TCR tg cells were incubated with peptide pulsed APC, 10 ng/ml IL-2 and various concentrations of chemical inhibitors that block phosphorylation of Jak3 or dimerization of STAT5a and STAT5b. Four days later, CD4 effectors were analyzed for GrB expression by flow cytometry after gating on CD4^+^/KJ126^+^ cells (**A**). Panel **B** shows the percent of maximum GrB MFI in CD4 effectors treated with concentrations of inhibitors shown in panel **A**. Effectors incubated with DMSO are shown as a diluent control for inhibitors. Shown is the average +/− SD for 3 separate experiments. Jak3i at 400 nM and STAT5i at 50 µM show statistically significant decreases in GrB MFI († p = .0006, *p = .0005). In **C** and **D**, CD4 effectors were generated as described from naive CD4 TCR tg cells from 3 separate mice and 4 days later, co-incubated with peptide pulsed targets in a 4 h killing assay. Panel **C** shows representative histograms of CD4 effectors incubated with IL-2 or IL-2 and Jak3i at 400 nM and Panel **D** is the average +/− SD of 3 individual effector preparations per group. Cytotoxicity by CD4 effectors treated with Jak3i is statistically lower than cytotoxicity by CD4 effectors generated with IL-2 alone (*p = 0.0014).

Because STAT5 phosphorylation was enhanced and sustained up to 96 h in culture in the presence of IL-2 and correlated with cytotoxicity, we wanted to determine if blocking the Jak3/STAT5 pathway would inhibit cytotoxicity. We used pharmacologic inhibitors that selectively block Jak3 (PF-956980) [33] or the SH2 domain of STAT5, preventing DNA binding activity [34]. Figure 3A (top) shows that the Jak3 inhibitor at 40 and 400 nM block IL-2 induced GrB expression in CD4 effectors. Similarly, the STAT5 inhibitor blocks GrB expression in CD4 effectors at 10 and 50 µM. However, STAT5 inhibitor at 10 and 50 µM does not diminish CD25 expression, indicating there is not a global downregulation of protein expression (Fig. S1A). Figure 3B shows percent of maximal GrB expression over a number of experiments in CD4 effectors treated with Jak3 or STAT5 inhibitors compared to treatment with DMSO, the solvent used for the inhibitors. Inhibiting either Jak3 or STAT5 significantly reduces GrB expression in CD4 effectors incubated with IL-2. To confirm that reduction of GrB expression correlated with killing activity, CD4 effectors were generated in the presence of IL-2 with and without Jak3 inhibitor at 400 nM for 4 days *in vitro*. Cytotoxicity was determined by staining for Annexin V on peptide pulsed target cells after incubation with CD4 effectors (Fig. 3C). Figure 3D shows the average of Annexin V^+^ cells cultured with CD4 effectors grown in IL-2 or in IL-2 and Jak3i and demonstrates that CD4 effectors generated in the presence of Jak3 inhibitor are markedly inhibited in their ability to kill peptide pulsed targets compared to CD4 effectors generated without inhibitor. Jak3 inhibitor does not induce cell death in CD4 effectors as measured by Annexin V^+^ staining in the cytotoxicity assay (Fig. S1B). Thus, generation of CD4 cytotoxicity requires phosphorylation and activation of Jak3 and STAT5.

### IL-2Rα (CD25) Deficiency Impairs Differentiation of CD4 T Cells into Cytolytic Effectors in vitro

**Figure 4:**
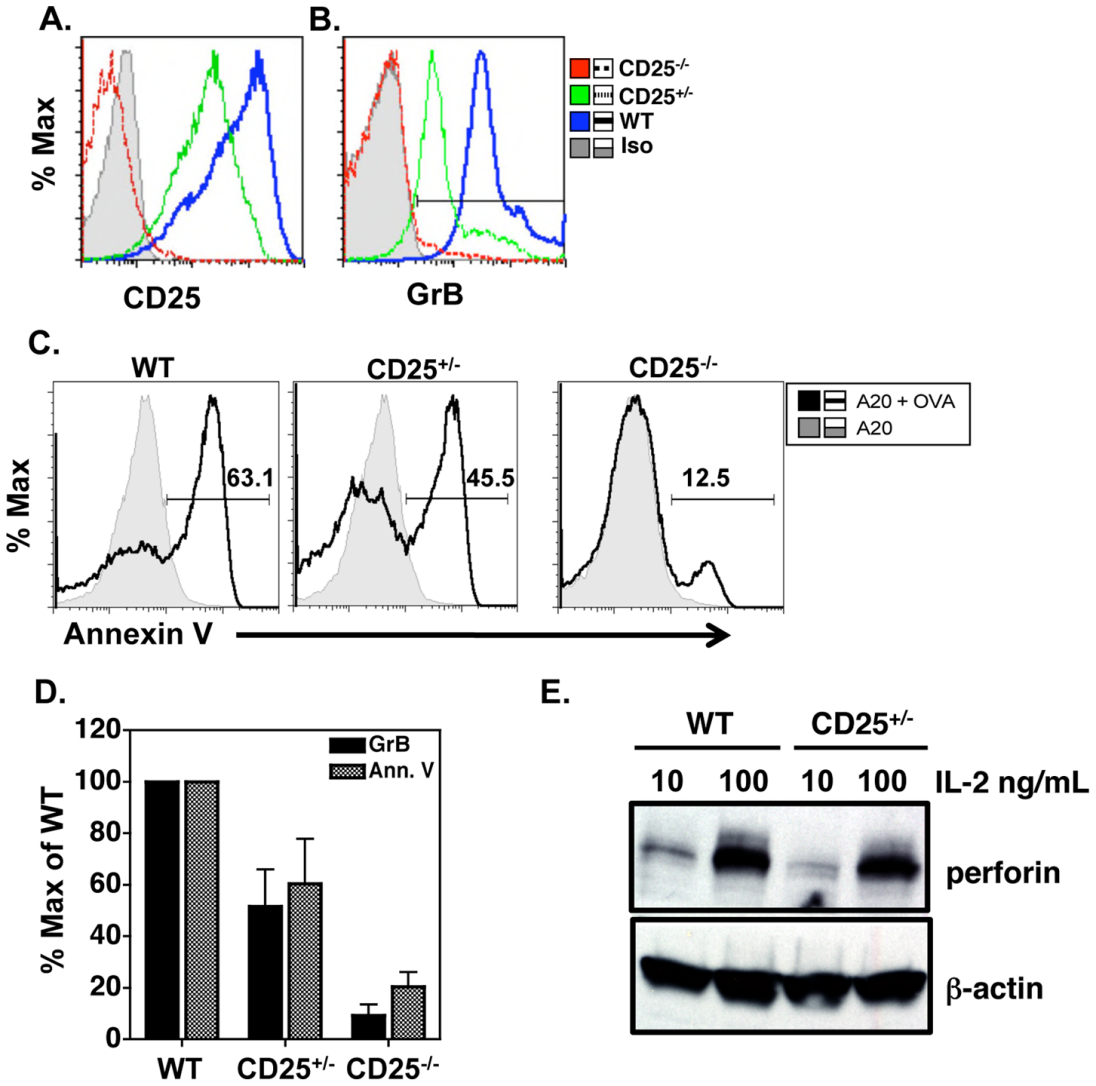
Expression of the high affinity IL-2R (CD25) is necessary for optimal CTL activity. Naive CD4 cells from DO11.10 (WT), DO11.10/CD25^+/−^ and DO11.10/CD25^−/−^ mice were cultured with Ova pulsed APC and IL-2 as described. Four days later, cells were collected and stained for CD4/KJ126, CD25 (**A**) and intracellular levels of GrB (**B**). Representative histograms are shown in Panel **A** and **B**. In **C**) 4 day WT, CD25^+/−^ and CD25^−/−^ effectors were collected and co-incubated with peptide coated A20 cells in a 4 h killing assay. Shown are representative histograms of percent Annexin V positive cells of peptide pulsed A20, using unpulsed A20 as a negative control to set histogram gates. Panel **D** is the average +/− SD of GrB intensity in different effector populations compared to Annexin V^+^ target cell killing for each effector population over 4 independent experiments. In each experiment, GrB MFI and Annexin V^+^ cells incubated with WT was used as 100% maximum value. **E**) Lysates of effectors generated with 10 or 100 ng/ml IL-2 were run on SDS-PAGE gel as described and incubated with antibody to perforin. Levels of β-actin are shown as a loading control.

Because IL-2, but not other common γ chain cytokines, could induce cytotoxicity in CD4 effectors we wanted to determine the contribution of the high affinity IL-2R (IL-2Rα, CD25) in driving the CD4 CTL phenotype. To assess the requirement for IL-2 receptor signaling, we generated CD4 effectors from wildtype (WT) Ova specific TCR tg mice, Ova specific IL-2Rα knockout (DO11.10/CD25^−/−^) mice as well as CD25^+/−^ littermates. WT CD4 effectors generated in the presence of 10 ng/ml IL-2 express high levels of IL-2Rα while CD25^+/−^ CD4 effectors express half the amount of IL-2Rα and CD25^−/−^ cells do not express IL-2Rα when activated (Fig. 4A). In agreement with previous results, WT DO11.10 cells express GrB and demonstrate peptide specific cytotoxicity (Fig. 4B and C). We also show that CD25 deficient cells do not express GrB (Fig. 4B) or acquire efficient cytolytic activity (Fig. 4C) when cultured in the presence of IL-2. Accordingly, CD25^+/−^ cells exhibit half maximal expression of GrB and display reduced cytotoxicity (Fig. 4B and C) when compared to WT cells. Figure 4D shows GrB expression levels and killing activity as a percent of wildtype, indicating the level of IL-2Rα expression correlates with cytolytic potential. Prf expression was also analyzed in WT and CD25^+/−^ CD4 effectors and shows that CD25^+/−^ cells express less Prf compared to WT cells after treatment with 10 ng/ml IL-2, however, addition of 100 ng/ml IL-2 increases Prf expression in CD25^+/−^ cells similar to WT (Fig. 4E). These results suggest the high affinity IL-2Rα is required for CD4 CTL generation *in vitro*. In addition, cytotoxicity is dependent on the amount of IL-2Rα present on CD4 cells (Fig. 4) as well as the amount of IL-2 in the culture (Fig. 1, Fig. 4), suggesting IL-2 signal strength impacts development of CD4 cytotoxicity.

### CD25 Deficient CD4 T Cells are Impaired in Effector Differentiation in vivo

Our previous studies demonstrated that cytolytic CD4 T cells differentiate *in vivo* following infection with highly pathogenic IAV, PR8 (H1N1). Influenza specific CD4 cells isolated from the lung, but not the draining lymph node (DLN), at 7 days post infection (dpi) expressed GrB and could lyse peptide coated targets directly *ex vivo* in a Prf dependent manner [13]. However, the signals required to direct the differentiation of naive CD4 cells into cytolytic effectors *in vivo* have not been elucidated. Given the role of IL-2 and IL-2Rα in the generation of cytolytic effectors *in vitro*, we next wanted to determine the contribution of IL-2Rα (CD25) in the generation of CD4 CTL *in vivo* in response to acute influenza infection.

**Figure 5:**
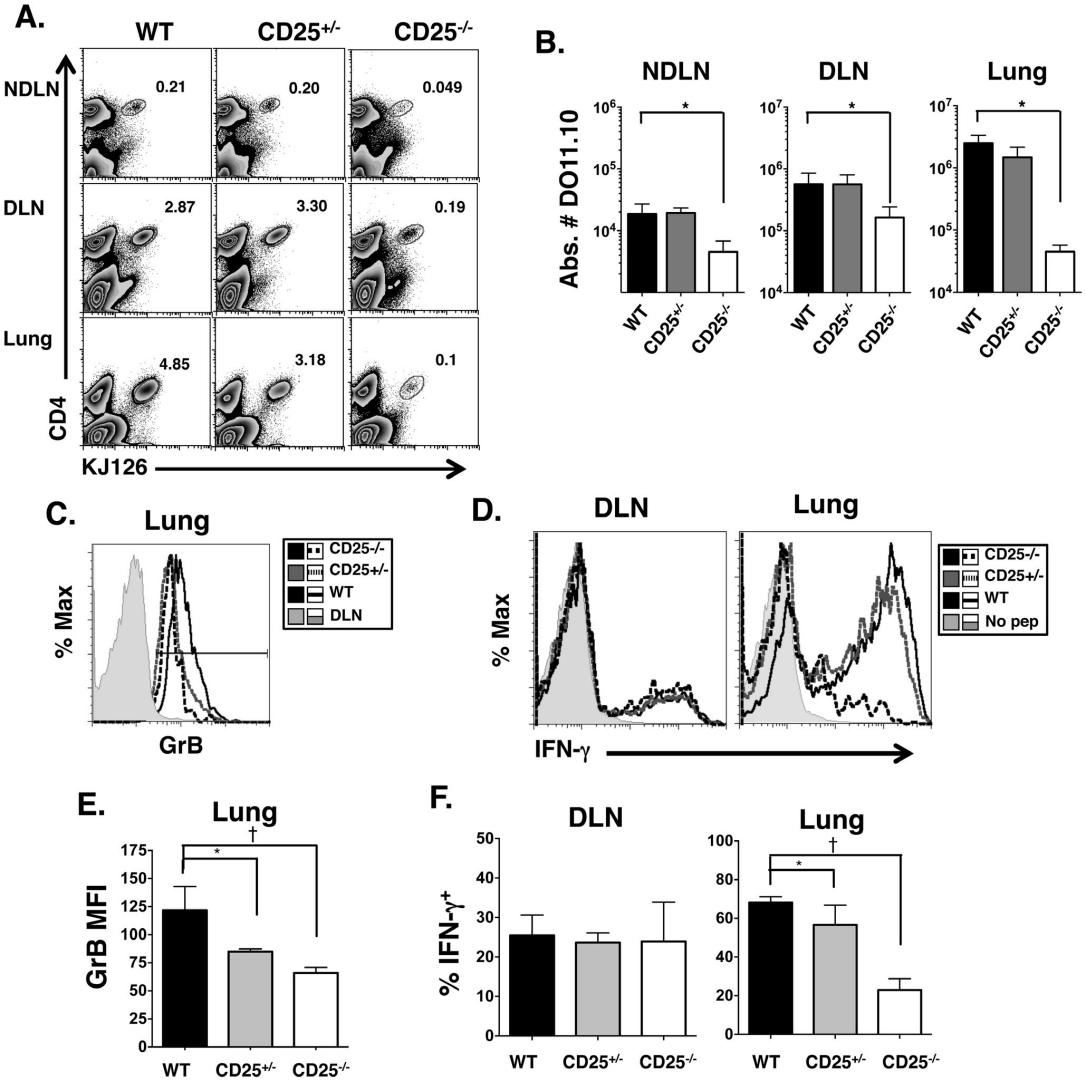
High affinity IL-2R is required for optimal GrB expression and IFN- γ **secretion in response to influenza infection.** Ova specific cells were transferred i. v. to BALB/c mice followed by infection with 5000 EID_50_ PR8/Ova. Seven dpi, mice were sacrificed, NDLN, DLN and lungs isolated and stained with antibodies to CD4 and Ova specific TCR (KJ126). **A**) Shown are representative FACS plots and percentage of Ova specific CD4 cells in NDLN (top) DLN (middle) and lung (bottom) samples. **B**) Total number of Ova specific CD4 cells were also calculated for each organ based on percentages from (**A**) and total cell numbers (p = .0002). Total lung cells were stained with CD4, KJ126 and intracellular stained for GrB directly *ex vivo*. Panel C shows a representative overlay histogram after gating on CD4^+^/KJ126^+^ cells and panel E shows the mean fluorescent intensity (MFI) of GrB expression for all mice. Total cells in the DLN and lung were restimulated with Ova_323-339_ peptide followed by intracellular staining for IFN-γ (D and F). **D**) Shown are representative overlay histograms of % IFN-γ cells in the DLN (left panel) or lung (right panel) after gating on CD4^+^/KJ126^+^ cells. Panel F shows the percent IFN-γ positive cells in the DLN (left panel) or lung (right panel) of 5 individual mice per group. The bar represents the average of all 5 mice per group. *p is <0.05 by student’s t test. ^†^ denotes p<.002 by student’s t test.

To address the requirement for IL-2 signaling, WT DO11.10, DO11.10/CD25^+/−^ or DO11.10/CD25^−/−^ CD4 T cells were adoptively transferred into normal BALB/c mice. Mice were subsequently infected with a sublethal dose of IAV PR8 that expresses Ova_323-339_ peptide (PR8/Ova) [35]. At 7 dpi, mice were sacrificed and DLN and lungs were removed. Single cell suspensions from these tissues were stained with anti-CD4 and anti-KJ126 (DO11.10) antibodies to mark donor populations (Fig. 5A) and total cell numbers were calculated (Fig. 5B). Given the role of IL-2 in T cell growth, we were not surprised to find that CD25 deficient cells did not expand well in the DLN (Fig. 5A and 5B). CD25 deficient cells also did not survive as well as WT cells in the non-draining lymph node (NDLN) (Fig. 5A and 5B). No significant differences were observed, however, between the number of CD25^+/−^ CD4 cells and the WT CD4 cells in the DLN (Fig. 5A–B), suggesting these cells were able to expand in response to antigen to a similar degree. CD25^−/−^ cell numbers in the lung were greatly diminished, with an approximately 50-fold decrease in the absolute number compared to WT (Fig. 5A–B). In contrast, there was an approximately 2-fold difference in the absolute number of CD25^+/−^ cells in the lung compared to WT, however, this was not statistically significant (Fig. 5A–B). Thus, CD25 deficiency impairs CD4 T cell numbers in the DLN and lung during influenza infection.

We next looked for expression of GrB directly *ex vivo* (Fig. 5C) and IFN-γproduction after restimulation with Ova peptide (Fig. 5D) in donor populations of CD4 T cells. CD4 T cells isolated from the lung but not the DLN at 7 dpi expressed GrB (Fig. 5C). This was similar for donor and host populations (data not shown). From *in vitro* data, we did not expect DO11.10/CD25^−/−^ cells to express GrB while DO11.10/CD25^+/−^ cells would display an intermediate amount of GrB expression when compared to WT and CD25^−/−^ T cells. Unexpectedly, nearly 100% of the donor CD4 T cells in the lung expressed GrB, regardless of their ability to respond to IL-2 (Fig. 5C). However, significant differences in the mean fluorescence intensity (MFI) of GrB were observed, where CD25^+/−^ T cells displayed intermediate expression of GrB and CD25 deficient cells expressed the lowest amount of GrB (Fig. 5C and 5E). CD4 T cells were also restimulated *ex vivo* with Ova peptide in order to assess the level of IFN-γproduced. In the DLN, approximately 20–30% of donor CD4 T cells secreted IFN-γ following restimulation, which was again independent of their ability to respond to IL-2. In the lung, nearly 70% of WT DO11.10 CD4 T cells expressed IFN-γ upon restimulation, while an average of 55% of CD25^+/−^ and only 25% of CD25^−/−^ CD4 T cells expressed IFN-γ (Fig. 5D and F). Similarly, the MFI of IFN-γ expression was significantly different between the three groups of donor cells (data not shown). In summary, these results indicate that CD25 is required *in vivo* for optimal CD4 T cell numbers in the DLN and the lung. Furthermore, maximum CD25 expression is required for maximal GrB and IFN-γ expression, but not for IL-2 expression (Fig. S2F).

### High IL-2R Expression is Required for GrB and Th1 Functions, but not for Eomes Expression and Degranulation

**Figure 6:**
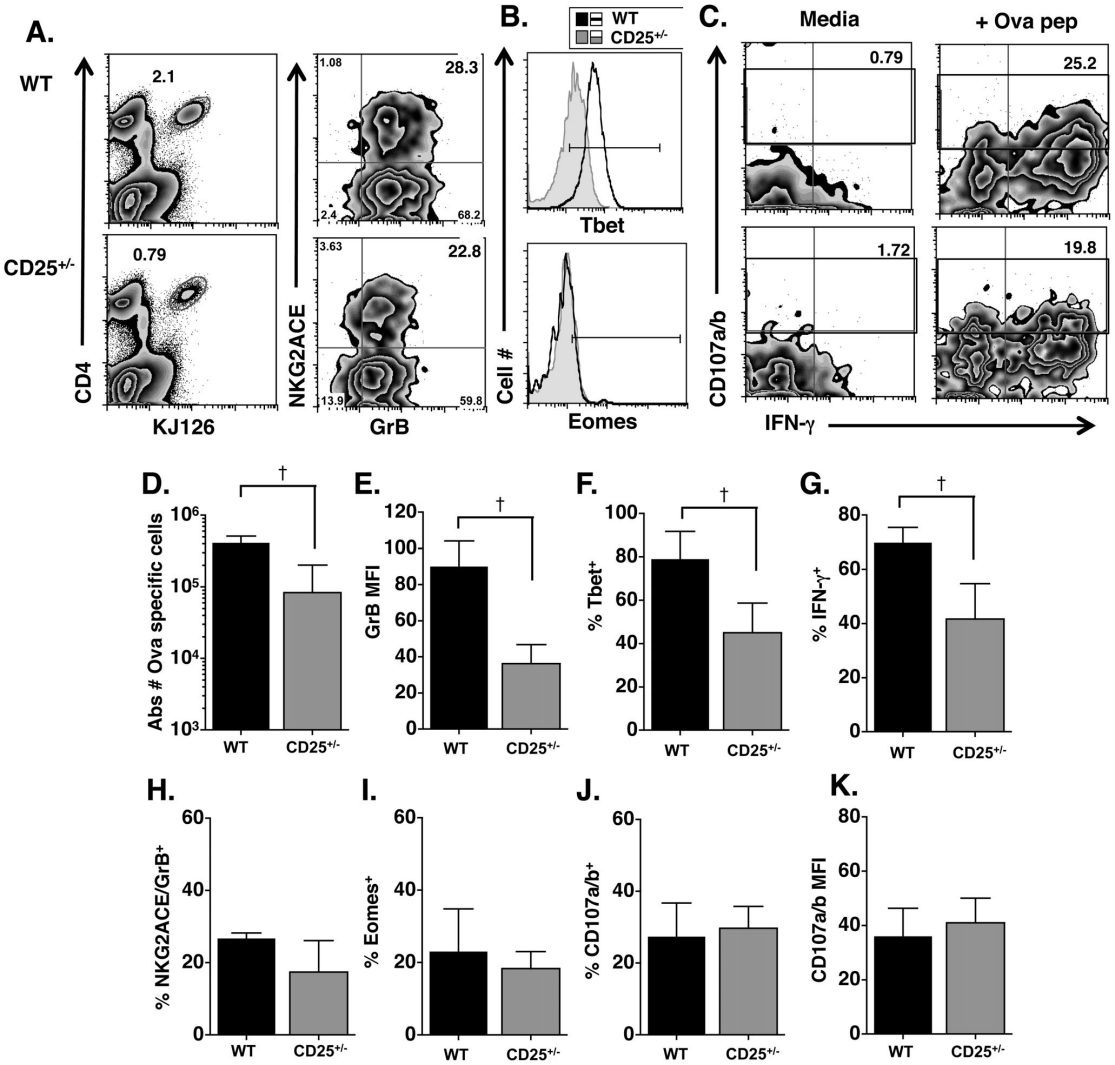
High IL-2R expression is required for GrB and Th1 functions, but not for cytolytic activity. Ova specific cells were transferred i. v. to BALB/c mice followed by infection with 1000 EID_50_ PR8/Ova. Seven dpi, mice were sacrificed, lungs isolated and stained with antibodies to CD4 and KJ126, followed by staining with NKG2A/C/E and intracellular GrB, or intracellular Tbet and Eomes. **A**) Shown are representative FACS plots and percentage of WT Ova specific CD4 cells (top) or CD25^+/−^ Ova specific CD4 cells (bottom). Also shown are representative 2 parameter histograms for NKG2ACE and GrB after gating on CD4^+^/KJ126^+^ cells. B) Representative overlay histograms of Tbet (top) and Eomes (bottom) after gating on Ova specific cells. The solid line represents expression in WT cells while the shaded histogram is expression in CD25^+/−^ cells. **C**) Lung cells were also restimulated with Ova peptide or media alone for 4h *in vitro* followed by staining for CD4, KJ126, CD107a/b and IFN-γ. Shown are 2 parameter histograms for CD107a and IFN-γ after gating on WT Ova specific cells (top) or CD25^+/−^ Ova specific cells (bottom) with or without (media) Ova peptide. The absolute number of Ova specific cells in lungs (**D**) is shown. GrB MFI (**E**), percent Tbet^+^ (**F**) and percent IFN-γ^+^ (**G**) after gating on Ova specific cells are shown. Percent NKG2A/C/E and GrB double positive cells (**H**), percent Eomes^+^ (**I**), percent CD107a/b^+^ cells (**J**) and CD107a/b MFI (**K**) are shown after gating on Ova specific cells. The † represents statistically significant differences where p<0.004 by student’s t-test.

While GrB has been used as a marker for cytolytic potential in CD4 effectors and is highly correlative with cytotoxicity [13], we have observed that high GrB expression does not necessarily indicate perforin mediated cytotoxicity ([13] and Workman, et al in preparation). Therefore, we wanted to test whether differences in IL-2Rα signaling led to differences in cytolytic activity in response to IAV infection. Because WT and CD25^+/−^ CD4 cells showed only a 2-fold difference in cell numbers in the lung, we compared Ova specific responses in WT and CD25^+/−^ effectors, but not CD25^−/−^ effectors after PR8/Ova infection. *In vivo* cytotoxicity assays [15], [28], [36] were initially employed to compare killing activity between WT and CD25^+/−^ effectors but demonstrated that CFSE labeled targets were lysed more efficiently in mice given WT DO11.10 cells than in mice given CD25^+/−^ cells (Fig. S3C). However, in this experiment, there was a much lower frequency of CD25^+/−^ cells in the lung compared to WT cells (Fig. S3A). Thus, it appeared that killing activity correlated with the number of Ova specific cells in the lung rather than the expression of cytolytic markers such as GrB. To analyze killing activity on a per cell basis, we used the degranulation assay, marked by appearance of CD107a/b on the cell surface of effector cells as degranulation occurs after restimulation with peptide pulsed target cells [11], [14], [24], [26], [37]. We also analyzed expression of NKG2A/C/E [38], [39] and intracellular expression of Eomesodermin [24]–[26] as additional markers of cytolytic potential in CD4 effector cells. Figure 6A and 6C indicate that CD25^+/−^ CD4 cells migrate to the lung at lower numbers than WT cells and demonstrate lower levels of GrB expression (Fig. 6A and 6D). In addition, CD25^+/−^ cells produced significantly lower levels of IFN-γ upon restimulation (Fig. 6B and 6F) as seen previously (Fig. 5F), and demonstrated lower Tbet expression (Fig. 6A and 6E). Surprisingly, NKG2A/C/E (Fig. 6A and 6G) and Eomes (Fig. 6A and 6H) expression was equivalent between WT and CD25^+/−^ CD4 cells. Further, the percent of DO11.10 cells degranulating, or expressing CD107a/b, was similar regardless of CD25 expression level (Fig. 6B and 6I). When the expression level of IFN-γ (data not shown) or CD107a/b (Fig. 6K) was measured by MFI, Th1 cytokine levels were decreased, but the amount of degranulation was similar between WT and CD25^+/−^ cells (Fig. 6J). Thus, IL-2 signaling appears to be necessary for Th1 cell functions such as IFN-γ production, but dispensable for degranulation, NKG2A/C/E and Eomes expression. In addition, high GrB expression is more correlative with Th1 functions than ability to degranulate and express Eomes.

## Discussion

IL-2 is a pleotropic cytokine that plays a complex role in CD4 cell expansion, survival and CD4 T helper subset generation. For example, IL-2 is absolutely required for Treg development [40]. IL-2 signaling is also necessary for effective Th1 development, where IL-2 enhances expression of IL-12Rβ2 and T-bet leading to high IFN-γ production [41]. At the same time, IL-2 can inhibit Th17 formation by downregulating gp130 and IL-6R [41]. Further work investigating the role of IL-2 signaling in CD4 T cells during IAV infection has shown that CD25 deficient CD4 cells differentiate more efficiently to the T follicular helper (Tfh) phenotype and suggests strong IL-2 signaling negatively impacts differentiation to Tfh [42]. Our previous work has shown that IL-2 positively regulates differentiation to a cytolytic phenotype in CD4 T cells [23]. These studies, however, did not address the amount of IL-2 needed to drive cytotoxicity, or the role of IL-2 signaling in driving CD4 CTL differentiation *in vivo*. Therefore, this paper set out to determine the relative contribution of IL-2 signaling to the development of CD4 CTL *in vitro* and *in vivo* in response to acute viral infection.

In this report, we show that the minimum requirements to drive GrB and killing activity in CD4 T cells are peptide pulsed APC and IL-2 (Fig. 1). However, while GrB expression was maximal at a relatively low dose of IL-2, high Prf expression required higher doses of IL-2 (Fig. 1C and 1D). This is similar to reports investigating the regulation of GrB and Prf expression in CD8 cells where strong IL-2R signals increased STAT5 and Eomes binding to the Prf promoter, thus enhancing Prf transcription [43]. In contrast, GrB expression in CD8 cells activated *in vitro* was not enhanced at higher IL-2 concentrations and was equally expressed at low and high dose IL-2 [43], similar to what we have shown here for CD4 effectors. Additionally, our data also indicate a role for Jak3 and STAT5 signals in driving GrB and cytotoxicity (Fig. 2). Although IL-15 can induce perforin expression in NK cells [44] and CD8 cells [45], and during priming of CD8 T cells [46], our results indicate that IL-15 induces only moderate upregulation of GrB and cytotoxicity in CD4 cells after TCR stimulation (Figure 2C and D). The contribution of Jak3 and STAT5 signaling in driving GrB and killing was confirmed using selective inhibitors of these kinases. Thus, strong IL-2 signals, acting through Jak3 and STAT5 were necessary to induce GrB, Prf and cytotoxicity in CD4 effectors. Because of the differential expression of IL-2 in the DLN vs lung after influenza infection, we suggest that initial signals through IL-2R and possibly IL-12R delivered upon antigen recognition in the DLN are necessary to program early CD4 CTL differentiation while inflammatory signals in the lung such as IL-6 (Workman, et al in preparation) and type I IFNs [27] enhance perforin expression and killing. The intracellular Jak/STAT signals that control enhanced CD4 CTL effector function have yet to be determined in this system.

The role for IL-2 signaling in generation of CD4 CTL was further supported in studies examining the contribution of the high affinity IL-2 receptor. We show here that CD25 expression had a positive impact on GrB and Prf expression as well as cytotoxicity *in vitro*. Again, strength of IL-2 signaling was important in driving Prf expression since CD25^+/−^ CD4 cells cultured in low dose IL-2 showed less Prf expression than WT CD4 cells, but the defect in CD25 was overcome by high dose IL-2 (Fig. 4E).

We next tested the contribution of CD25 in the generation of CD4 CTL *in vivo* in response to acute influenza infection. Our results show that *in vivo*, CD25 deficiency impacts GrB expression (Fig. 5C and E), IFN-γsecretion in the lung (Fig. 5D and F), and number of CD4 cells in the lung (Fig. 5A and B). It has been demonstrated that IL-2 regulates many T cell trafficking genes [47]. However, the lower number of CD25^−/−^ CD4 cells in the lung could be due to less expansion in the DLN (Fig. 5B) or less survival overall since CD25^−/−^ cells are also significantly decreased in the NDLN (Fig. 5A). In any case, the work described herein and our previous data [13] point to a role for the lung microenvironment in supplying additional signals to drive cytotoxicity. First, CD4 cells in the DLN do not express GrB or demonstrate killing activity [13]; second, IL-2R deficiency did not impact IL-2 and IFN-γ production in the DLN (Fig. 5F, Fig. S2), but did impact CD4 effector functions in the lung. Thus, we hypothesize that CD4 CTL receive secondary signals in the lung to drive GrB and killing activity. Indeed, it has been shown that CD8 CTL require secondary signals from dendritic cells in the lung for protective efficacy [48] and IL-15 presented in trans was necessary for enhanced survival of CD8 CTL [49] during IAV infection. Whether CD4 CTL require inflammatory cytokines such as IL-6 in the lung, or interact with lung resident DCs that may present IL-2 or IL-15 in trans [50] are future questions to be answered.

While GrB has been used as a marker for cytolytic potential in CD4 CTL, our preliminary data suggests that high GrB expression does not always correlate with perforin-mediated cytotoxicity (Workman et al., in preparation). Therefore, we also analyzed expression of NKG2A/C/E and Eomes and measured degranulation by the appearance of CD107a/b as additional markers of cytolytic potential following influenza infection. These studies demonstrate that the ability to degranulate and express NKG2A/C/E and Eomes was independent of high IL-2R expression. The percentage of cells expressing GrB was also independent of IL-2R expression (Fig. 5C, 6H, Fig. S2). In contrast, the amount of GrB, as measured by MFI, was dependent on IL-2Rα levels (Fig. 5E, 6E). Similarly, measures of Th1 differentiation, such as IFN-γ secretion and Tbet expression, were also dependent on IL-2Rα (Fig. 5F, 6F, 6G). This is slightly different that what has been observed in CD8 T cells. In the case of CD8 T cells, Eomes expression was highly dependent on IL-2 *in vitro* [43]. Further results showed that CD25^−/−^ CD8 T cells exhibited lower lytic ability, lower Prf expression by qPCR and lower levels of GrB and KLRG1, but similar peptide specific IFN-γ production [43]. Thus, these results highlight the role of IL-2 in driving CD4 effector functions in which IL-2R signaling plays a major role in Th1 differentiation but may be dispensable for CD4 CTL functions, especially in the context of infection. Indeed, recent work by Hua et al point to a role for type I IFN in driving full CD4 CTL activity in response to influenza infection [27]. Our data suggests that IL-2 signals in the DLN may prime CD4 CTL for receiving additional cytokine signals in the lung microenvironment for optimal perforin expression and CTL activity. Preliminary data indicates that higher pathogenicity IAV induces higher levels of IL-6 in the lung microenvironment and influx of CD4 effectors with greater cytolytic potential than lower pathogenicity IAV strains (Workman, et al, in preparation). IL-6 has been shown to enhance perforin mediated cytolytic activity in CD8 cells [51], thus, this inflammatory mediator may also have direct effects on enhancing perforin mediated cytotoxicity in lung resident CD4 CTL.

In summary, this work begins to delineate the signals required for the differentiation of CD4 T cell effectors with cytolytic potential. *In vitro*, IL-2 signaling via STAT5 activation was required to induce GrB and cytotoxicity in CD4 effectors. Amount of IL-2 and IL-2Rα levels regulated GrB and perforin expression leading to maximal CD4 CTL differentiation *in vitro*. *In vivo*, high IL-2Rα expression was necessary for maximal GrB expression and Th1 effector functions in response to IAV infection. However, inflammation induced by viral infection could overcome the requirement for high CD25 expression in inducing NKG2A/C/E, Eomes and degranulation in antigen specific T cells, suggesting that cytolytic potential and Th1 effector potential may be differentially regulated. It is still unclear whether CD4 CTL represent a terminally differentiated Th1 effector [7], [9], comprise a novel T helper subset [52], or differentiate in the same way as CD8 CTL [53]. In tumor studies, high levels of costimulation with OX-40/OX-40L [24], 4-1BB [25] or costimulation in conjunction with lymphopenia [26] induce a subset of CD4 CTL that express high levels of the T-box transcription factor, Eomes. Recent data indicates that CD4 CTL generated during viral infection express Blimp-1 and T-bet transcription factors, but not Eomes [27]. More work is necessary to determine whether anti-viral CD4 CTL develop through a separate T helper pathway. As CD4 CTL have been shown to be important in influenza, HIV and anti-tumor responses, this information will be vital for inducing potent CD4 effectors with multifunctional properties against a variety of diseases.

## Materials and Methods

### Mice

TCR tg mice in which CD4 cells recognize Ova_323-339_ on the BALB/c background (DO11.10) as well as DO11.10 TCR tg mice deficient in the ability to produce IL-2 (IL-2^−/−^) or the IL-2Rα subunit (CD25^−/−^) were a generous gift from Dr. Abul Abbas (UCSF) and used at 6–8 weeks of age. BALB/c By mice were purchased from The Jackson Laboratory, Bar Harbor, ME and used at 8–10 weeks of age.

### Ethics Statement

These experiments were performed in accordance with the recommendations in the Guide for the Care and Use of Laboratory Animals of the National Institutes of Health. Our protocol was approved by the Institutional Animal Care and Use Committee of the University of Nebraska-Lincoln, protocol #695-E on 6/24/2011. All efforts were made to minimize suffering of animals.

### Medium, Peptides, Cytokines and Virus

All cells were grown in RPMI 1640 media (Invitrogen, Carlsbad, CA) containing 2 mM L-glutamine, 100 IU penicillin, 100 µg/ml streptomycin (all from Invitrogen) 10 mM HEPES (Sigma, St. Louis, MO), 50 µM 2-mercaptoethanol (Sigma) and 8% fetal bovine serum (FBS) (Hyclone, Logan, UT). Ova peptide_323-339_, ISQAVHAAHAEINEAGR was synthesized by New England Peptide, Inc. (Gardner, MA). Influenza virus A/Puerto Rico/8/34 (H1N1) expressing Ova_323-339_ peptide fragment (PR8/Ova) [35] was a generous gift from Dr. Paul G. Thomas (St. Jude Children’s Research Hospital, Memphis TN). Mouse IL-2 was purchased from PeproTech (Rocky Hill, NJ). Janus activating kinase (Jak)-3 inhibitor (PF-956980), purchased from Sigma and STAT5 inhibitor (N’-((4-Oxo-4H-chromen-3-yl)methylene) nicotinohydrazide, purchased from EMD Millipore Chemicals (Billerica, MA) were used at the indicated concentrations.

### Isolation of CD4 T Cells and in vitro Activation

CD4 effectors were generated *in vitro* as previously described [23], [28], with modifications listed below. Briefly, naive CD4 cells were isolated from TCR tg mice using a positive selection protocol with anti-CD4 beads (clone GK1.5) according to the manufacturer’s instructions (Miltenyi Biotech, Auburn CA). A20s (H-2^d^ expressing mouse B lymphoma cell line) were pulsed with Ova peptide_323-339_ at a concentration of 0.5 µM and combined with naive CD4 cells at a 1∶1 ratio. For IL-2 dose response studies, IL-2 was added to culture medium at concentrations ranging from 1 ng/ml to 100 ng/ml. After 2 days in culture, an equal volume of complete media containing the appropriate concentration of fresh IL-2 was added to all cultures that were then incubated for an additional 2 days.

On day 4, cultures were assayed for purity, activation and polarization as described previously [23], [28]. To assay for purity of the resulting TCR tg effector populations, CD4 cells were stained with PerCP labeled anti-CD4 (clone RMA-4.4) and FITC labeled antibodies to KJ126 (DO11.10) and activation state of effector populations was further assessed by staining cells with PE labeled antibodies to CD62L (MEL-14), CD43 (1B11), CD27 (LG.3A10) and CD25 (3C7) (all antibodies purchased from eBiosciences, San Diego, CA). Following surface staining, cells were fixed in 4% paraformaldehyde and incubated in saponin buffer (PBS plus 1% FBS, 0.1% NaN3 and 0.25% saponin; Sigma-Aldrich) containing APC labeled mouse anti-human GrB (Clone GB11, Invitrogen). APC conjugated mouse IgG1 was used as an isotype control. Cells were then analyzed using a Becton Dickinson FACS Caliber and data processed using Flow Jo software (Treestar, San Carlos, CA).

### Analysis of STAT5 Phosphorylation

To determine STAT5 activation in CD4 effectors, naive CD4 cells were incubated with peptide pulsed A20 cells and IL-2, IL-7 or IL-15. At various time points, cells were collected and stained with anti-CD4, anti-TCR (KJ126) and anti-CD44, followed by intracellular staining for phosphorylated STAT5 using anti-STAT5 (pY694) from BDBiosciences (San Diego, CA) as described in [54].

### In vitro Cytotoxicity Assay

For flow cytometric killing assays, A20 target cells were loaded with 5 uM Ova peptide for 1 hour prior to a 4 h co-incubation with CD4 effectors at a 3∶1 effector to target ratio. Following a 4 h incubation at 37°C, cell death was assayed using the Annexin V PE apoptosis detection kit (eBiosciences) as described by the manufacturer. Peptide specific lysis was determined relative to the spontaneous lysis control (CD4 effectors+targets *without* peptide). In some experiments the JAM assay was used to determine cell killing as previously described [23], [28]. Lytic units were then calculated from the JAM assay results as the number of lymphocytes required to yield 30% lysis in a population of 10^6^ cells [23].

### SDS-PAGE and Western Blot

*In vitro* generated CD4 effectors were washed with phosphate-buffered saline (PBS) and resuspended in RIPA buffer (Millipore, Temecula, CA) at 1×10^7^ cells/100 uL. Cell lysate was incubated on ice for 30 min and clarified by centrifugation at 14,000 rpm at 4°C for 15 min. Protein concentrations were quantified by the Bradford assay. For sodium dodecyl sulfate-polyacrylamide gel electrophoresis (SDS-PAGE), proteins were mixed with an equal amount of 1x sample loading buffer (62.5 mM Tris-HCl [pH 6.8], 2% SDS, 50 mM dithiothreitol, 0.1% bromophenol blue, 10% glycerol) and boiled for 5 min. Proteins were separated in a 7.5% SDS-PAGE gel. After electrophoresis, proteins were transferred onto a polyvinylidene difluoride membrane (Immobilon-P; Millipore) and blocked for 2 h in 5% nonfat dry milk with Tris-buffered saline–0.1% Tween 20 (TBS-T). Membranes were then incubated with primary antibody overnight at 4°C. The anti-perforin antibody clone CB5.4 (Abcam, Cambridge, MA) was diluted 1∶1000 in the blocking solution. An antibody directed against β-actin (Abcam) was used as a loading control. After 45 min of washing with TBS-T, the blots were incubated with goat anti-rat (Abcam) or goat anti-rabbit (R&D Systems, Minneapolis MN) HRP-conjugated IgG, which was diluted in blocking solution. Following 1 hour incubation at room temperature, blots were washed for 45 min with TBS-T, exposed to Amersham ECL reagents, and autoradiography was performed.

### Adoptive Transfer and Infection of Mice

1×10^6^ naïve CD4 T cells (isolated as described above) were adoptively transferred into normal BALB/c By mice via retro orbital injection. Eighteen to 24 hours after CD4 T cell injection, mice were infected intranasally with 1,000 or 5,000 EID_50_ of PR8/Ova virus [35].

### Isolation of Cell Populations for FACS Analysis of Donor and Host Populations

PR8/Ova infected BALB/c mice were sacrificed at 7 days post infection (dpi) and lungs perfused via the right ventricle of the heart with PBS, minced and incubated with collagenase D (2.5 mg/ml final concentration) at 37°C for 75 minutes to dissociate lung tissue. Single cell suspensions were generated by passing lung homogenate through a 70 µM mesh filter. Draining lymph nodes (DLN) were dissociated through a 70 µM mesh filter to generate single-cell suspensions. Single cell suspensions from these tissues were stained with PerCP labeled anti-CD4 (clone RMA-4.4) and FITC labeled anti-DO11.10 TCR (KJ126) for 30 min in FACS buffer at 4°C to mark donor cells. Lung cells were subsequently fixed in 100 µl 4% paraformaldehyde and stained for intracellular GrB using APC labeled mouse anti-human GrB (GB11) in saponin buffer for 40 minutes at room temperature.

Cells from DLN or lung were also restimulated for 4 h with peptide-pulsed A20 cells (2.5 µg/ml Ova peptide) as APCs. 10 µg/ml Brefeldin A (Sigma) was added for the final 2 h of culture and maintained throughout the intracellular cytokine staining procedure. Donor T cells were surface stained with FITC labeled antibodies to KJ126 (DO11.10) and PerCP conjugated anti-CD4 as described previously, fixed in 100 µl 4% paraformaldehyde, and stained in saponin buffer containing anti–IFN-γ APC (BD-Biosciences) and IL-2 PE. In some experiments, lung cells were restimulated with Ova peptide pulsed or unpulsed A20 cells in the presence of anti-CD107a-PE and anti-CD107b-PE for 4 h at 37°C. Brefeldin A was added for the last 2 h in culture, followed by intracellular staining for IFN-γ as described. Lung cells restimulated with CD8 specific NP peptide were used a positive control for degranulation. Cells were then analyzed using a Becton Dickinson FACS Caliber and data processed using Flow Jo software (Treestar, San Carlos, CA).

### Statistical Analyses

Statistical analyses were performed using Prism software. Students two-tailed unpaired t-test was used for analysis of statistical significance between experimental samples.

## Supporting Information

Figure S1 **Pharmacological inhibitors of the Jak/STAT pathway do not inhibit CD25 expression or induce high levels of cell death in CD4 effectors.** Naive CD4 cells were activated *in vitro* with peptide pulsed APC and IL-2 in the presence or absence of inhibitors. A) Levels of CD25 expression in CD4 effectors analyzed 4 days after culture with STAT5 inhibitors. These same effectors were analyzed for GrB expression as shown in Figure 3A. B) Analysis of CD4 effector viability by Annexin V staining after 4h in culture with peptide pulsed A20 target cells after gating on CD4^+^ population. Effectors generated in the presence of Jak3 inhibitor did not demonstrate increased apoptosis, although CD4 effectors incubated with peptide pulsed targets showed increased apoptosis. These same effectors were analyzed for killing activity in Figure 3D.
(TIF)

Figure S2 **CD4 T cells deficient in IL-2Rα show defects in GrB and IFN-γ, but not IL-2, production at 1000 EID_50_ PR8/Ova infection.** Mice were adoptively transferred with WT, CD25^+/−^ and CD25^−/−^ Ova specific CD4 T cells as described and subsequently infected with 1000 EID_50_ PR8/Ova i. n. Seven days p. i., mice were sacrificed, lungs removed and cells stained with antibodies to CD4 and Ova specific TCR (KJ126). A) Shown are representative FACS plots and percentage of Ova specific CD4 cells in lung samples. B) Total lung cells were stained with CD4, KJ126 and intracellular stained for GrB directly *ex vivo*. Panel B shows a representative overlay histogram after gating on CD4^+^/KJ126^+^ cells and panel D shows the mean fluorescent intensity (MFI) of GrB expression for all mice. C) Total number of Ova specific CD4 cells was also calculated for the lung based on percentages from (A) and total cell numbers (p>.002). Total cells in the lung were restimulated with Ova_323-339_ peptide followed by intracellular staining for IFN-γ (E) or IL-2 (F). Shown is the average +/− SD percent IFN-γ positive cells (E) or percent IL-2 positive cells (F) in the lung of 5 individual mice per group. *p is <0.05 by student’s t test.
(TIF)

Figure S3 **WT CD4 cells demonstrate enhanced killing *in vivo* that correlates with frequency of cells in the lung.** WT or CD25^+/−^ DO11.10 were adoptively transferred to BALB/c mice followed by infection with PR8/Ova virus. Seven dpi, naive Ova_323-339_ pulsed CD19^+^ cells were labeled with 5 µM CFSE and combined at a 1∶1 ratio with unpulsed CD19^+^ cells labeled with 0.5 µM CFSE and injected i. v. Eighteen hours after target injection, mice were sacrificed, spleens were removed, red cells were lysed and resuspended in FACS buffer. Cells were analyzed with a BD Biosciences FACSCalibur, and data were processed using FlowJo software (Tree Star). Percentage of specific cytotoxicity was calculated as follows: 100– {((percentage of peptide pulsed in transferred/percentage of unpulsed in transferred)/(percentage of peptide pulsed in naive/percentage of unpulsed in naive))×100}. Panel **A** shows the percentage of Ova specific cells in the DLN and lung 7 dpi while panel **B** shows the level of GrB expression in Ova specific lung cells. Panel **C** is the calculated % cytotoxicity after analysis of CFSE labeled targets in the spleen.
(TIF)
