# Supplementary figures and images for: Inflammation Enhances IL-2 Driven Differentiation of Cytolytic CD4 T Cells
Source: PLoS One. 2014 Feb 20;9(2):e89010. doi: 10.1371/journal.pone.0089010 (PMC3930678; doi:10.1371/journal.pone.0089010)

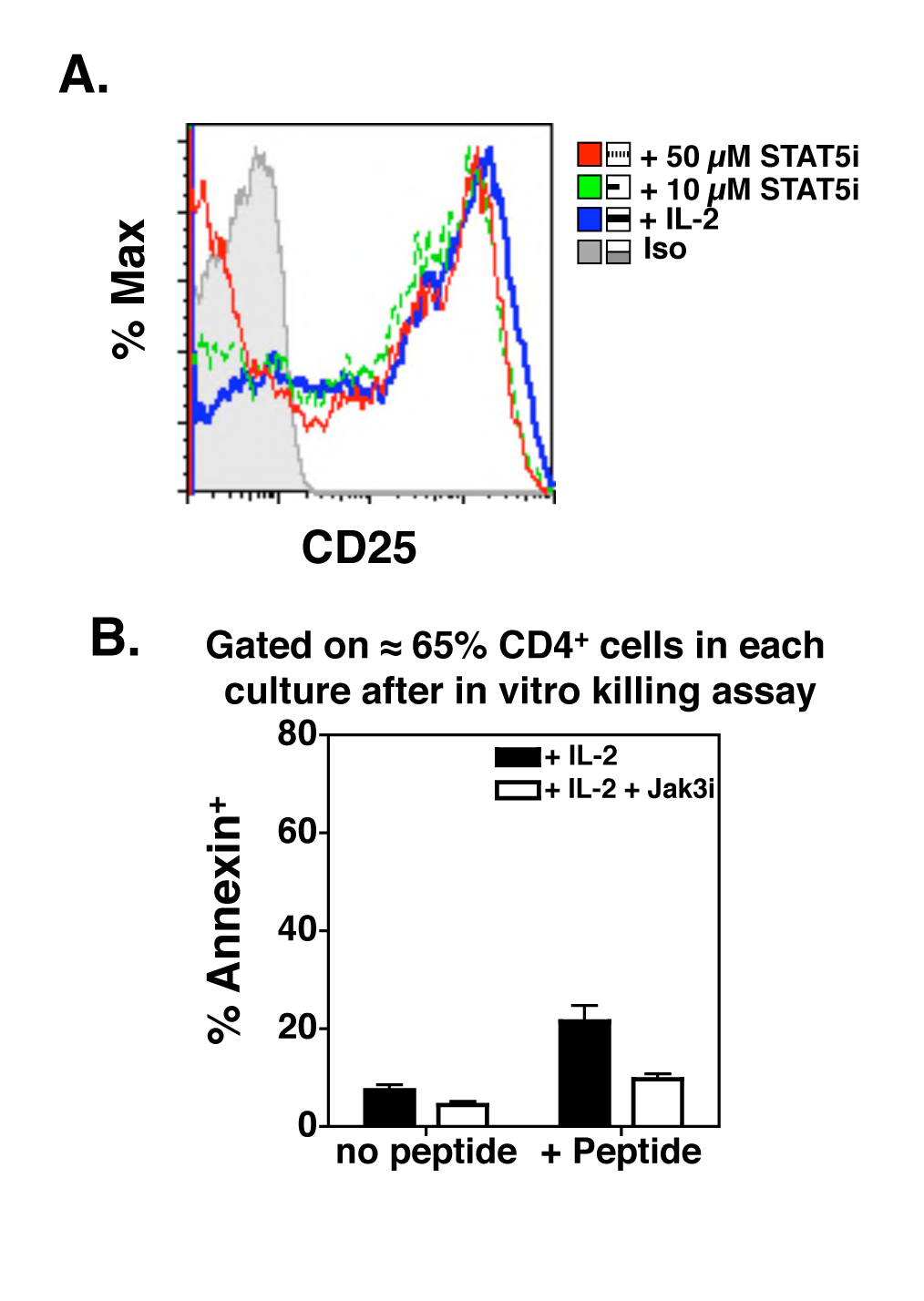

Supplement: Figure S1 — Pharmacological inhibitors of the Jak/STAT pathway do not inhibit CD25 expression or induce high levels of cell death in CD4 effectors. Naive CD4 cells were activated in vitro with peptide pulsed APC and IL-2 in the presence or absence of inhibitors. A) Levels of CD25 expression in CD4 effectors analyzed 4 days after culture with STAT5 inhibitors. These same effectors were analyzed for GrB expression as shown in Figure 3A. B) Analysis of CD4 effector viability by Annexin V staining after 4h in culture with peptide pulsed A20 target cells after gating on CD4+ population. Effectors generated in the presence of Jak3 inhibitor did not demonstrate increased apoptosis, although CD4 effectors incubated with peptide pulsed targets showed increased apoptosis. These same effectors were analyzed for killing activity in Figure 3D. (TIF) [file pone.0089010.s001.tif]

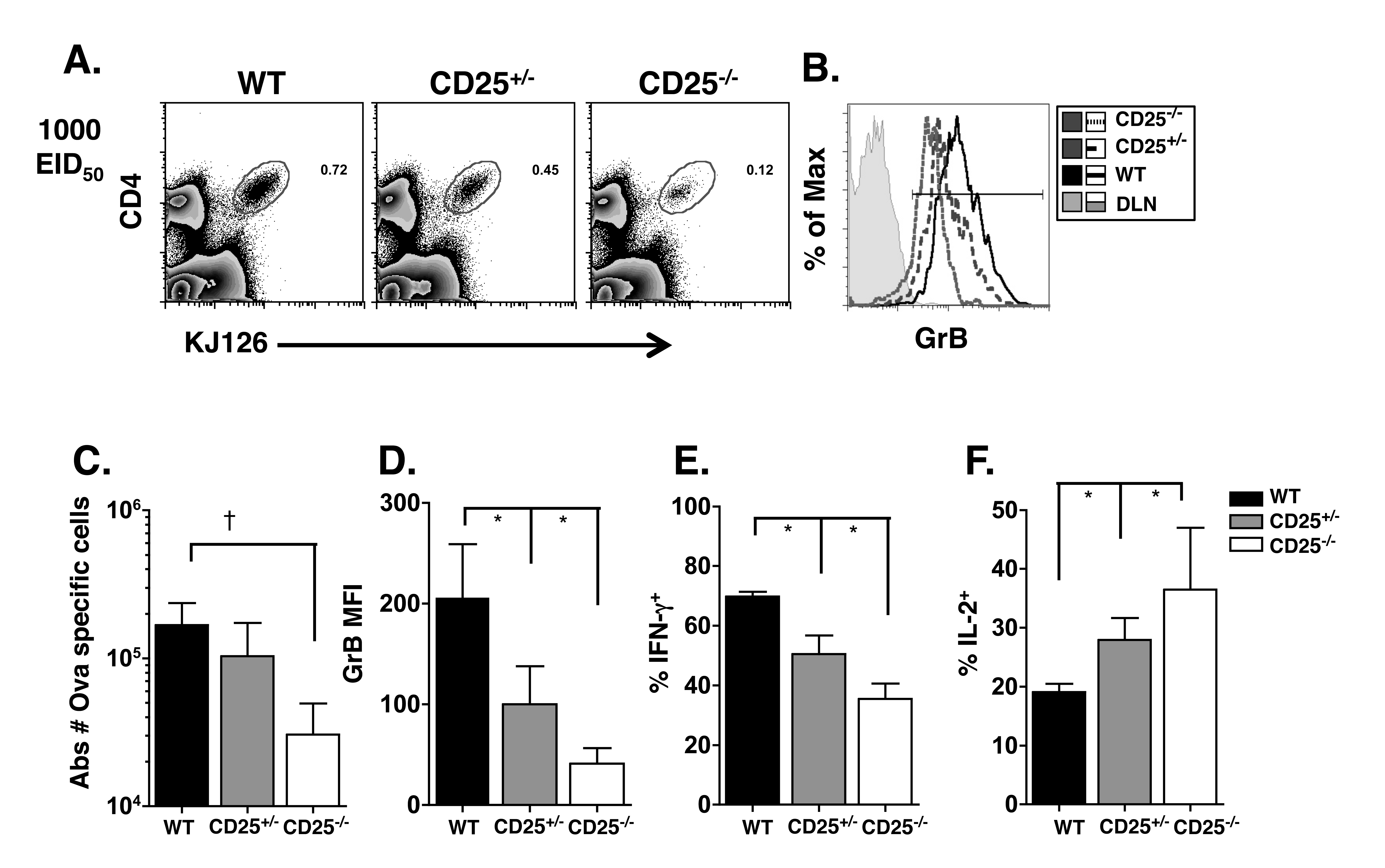

Supplement: Figure S2 — CD4 T cells deficient in IL-2Rα show defects in GrB and IFN-γ, but not IL-2, production at 1000 EID50 PR8/Ova infection. Mice were adoptively transferred with WT, CD25+/− and CD25−/− Ova specific CD4 T cells as described and subsequently infected with 1000 EID50 PR8/Ova i. n. Seven days p. i., mice were sacrificed, lungs removed and cells stained with antibodies to CD4 and Ova specific TCR (KJ126). A) Shown are representative FACS plots and percentage of Ova specific CD4 cells in lung samples. B) Total lung cells were stained with CD4, KJ126 and intracellular stained for GrB directly ex vivo. Panel B shows a representative overlay histogram after gating on CD4+/KJ126+ cells and panel D shows the mean fluorescent intensity (MFI) of GrB expression for all mice. C) Total number of Ova specific CD4 cells was also calculated for the lung based on percentages from (A) and total cell numbers (p>.002). Total cells in the lung were restimulated with Ova323-339 peptide followed by intracellular staining for IFN-γ (E) or IL-2 (F). Shown is the average +/− SD percent IFN-γ positive cells (E) or percent IL-2 positive cells (F) in the lung of 5 individual mice per group. *p is <0.05 by student’s t test. (TIF) [file pone.0089010.s002.tif]

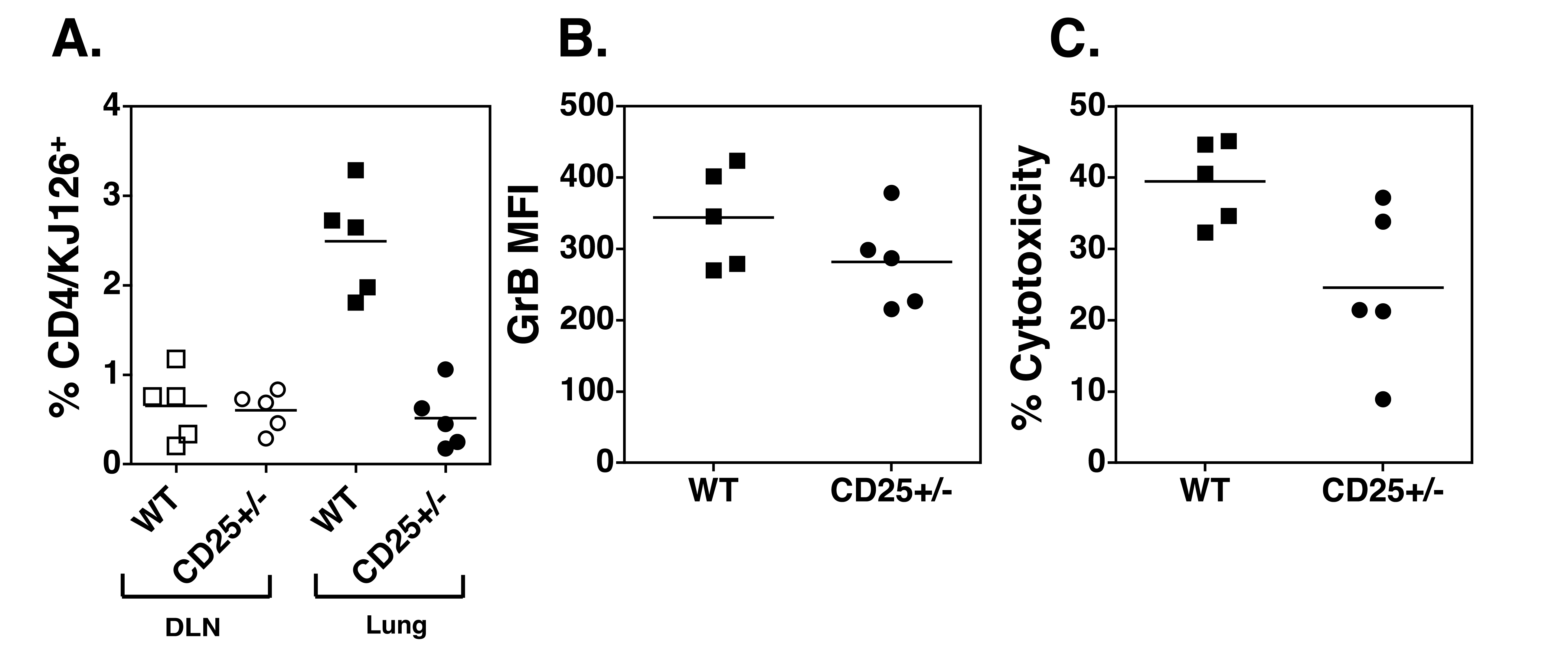

Supplement: Figure S3 — WT CD4 cells demonstrate enhanced killing in vivo that correlates with frequency of cells in the lung. WT or CD25+/− DO11.10 were adoptively transferred to BALB/c mice followed by infection with PR8/Ova virus. Seven dpi, naive Ova323-339 pulsed CD19+ cells were labeled with 5 µM CFSE and combined at a 1∶1 ratio with unpulsed CD19+ cells labeled with 0.5 µM CFSE and injected i. v. Eighteen hours after target injection, mice were sacrificed, spleens were removed, red cells were lysed and resuspended in FACS buffer. Cells were analyzed with a BD Biosciences FACSCalibur, and data were processed using FlowJo software (Tree Star). Percentage of specific cytotoxicity was calculated as follows: 100– {((percentage of peptide pulsed in transferred/percentage of unpulsed in transferred)/(percentage of peptide pulsed in naive/percentage of unpulsed in naive))×100}. Panel A shows the percentage of Ova specific cells in the DLN and lung 7 dpi while panel B shows the level of GrB expression in Ova specific lung cells. Panel C is the calculated % cytotoxicity after analysis of CFSE labeled targets in the spleen. (TIF) [file pone.0089010.s003.tif]
